# Willingness to pay for antiretroviral drugs among HIV and AIDS clients in south‐east Nigeria

**DOI:** 10.1111/hex.12612

**Published:** 2017-08-14

**Authors:** Chinyere Mbachu, Chijioke Okoli, Obinna Onwujekwe, Fabian Enabulele

**Affiliations:** ^1^ Health Policy Research Group Department of Pharmacology and Therapeutics College of Medicine University of Nigeria Enugu Campus Enugu Nigeria; ^2^ Department of Community Medicine University of Nigeria Enugu Campus Enugu Nigeria; ^3^ Department of Health Administration and Management College of Medicine University of Nigeria Enugu Campus Enugu Nigeria; ^4^ College of Health Sciences Ebonyi state University Abakaliki Nigeria

**Keywords:** antiretroviral drugs, clients, HIV and AIDS, willingness to pay

## Abstract

**Background:**

The current trend of withdrawal of donor support for HIV/AIDS treatment in Nigeria may require that the cost of antiretroviral drugs be borne in part by infected people and their families.

**Objective:**

This study was conducted to determine the economic value for free antiretroviral drugs (ARVs) expressed by clients receiving treatment for HIV/AIDS in a tertiary hospital.

**Study method:**

The contingent valuation method was used to elicit the values attached to free ARVs from people living with HIV/AIDS that were receiving care in a public tertiary hospital in south‐east Nigeria. Exit poll using a pre‐tested questionnaire was undertaken with adult clients on treatment. The bidding game technique was used to elicit their willingness to pay (WTP) for ARVs for themselves and members of their households. Ordinary least squares (OLS) multiple regression analysis was used to test the construct validity of elicited WTP amounts.

**Results:**

About a third of the respondents were willing to pay for a monthly supply of ARVs for themselves and household members. The mean WTP for monthly supply of ARVs for self was US$15.32 and for household member was US$15.26 (1US$=₦160). OLS regression analysis showed that employment status and higher socio‐economic status were positively associated with higher WTP. OLS showed that age and transport cost per clinic visit were negatively related to WTP. Knowing the risks of not adhering to treatment protocol was positively related to WTP.

**Conclusion:**

The respondents positively valued the free ARVs. This calls for greater financial support for the sustainable provision of the treatment service. However, holistic financing mechanisms should be explored to ensure sustained funding in the event of complete withdrawal of donor support.

## 1 INTRODUCTION

Africa bears the highest burden of HIV infections and HIV/AIDS related mortality in the world.^1^ HIV/AIDS is one of the most challenging health problems for policymakers in sub‐Saharan Africa.^2^ It poses a major threat to the economic viability of infected individuals and invariably reduces household income.^3^, ^4^, ^5^ Prevention and treatment of HIV/AIDS have, until recently, been driven solely by development partners and donor agencies, particularly the provision of antiretroviral drugs (ARVs).^2^, ^6^, ^7^ Nigeria operates a decentralized health system that is largely financed through tax‐based revenue and out‐of‐pocket spending.^8^, ^9^ Health‐care services in Nigeria are provided by a multiplicity of health‐care providers in the public and private sectors and access and utilization varies across socio‐economic and geopolitical subpopulation groups.^10^ Current estimates show that about one‐third of HIV‐infected people are on treatment with ART.^11^ HIV drugs are provided free of charge to clients through public and mission hospitals that offer at least secondary level of care. These types of facilities (also called comprehensive centres) are mostly found in urban areas, creating geographic inequities in access to HIV treatment.^12^

In the face of reducing donor assistance for health services delivery in Nigeria, programme managers will be tasked with the responsibility of making rational price decisions concerning services for which user fees were removed in the past. In order to ensure programme sustainability, it is important to maintain a balance between the social goal of making services available to low income clients and the survival of the programme over time.^13^, ^14^, ^15^ While it is clear that too high prices could result in financial inaccessibility for poor clients/patients, sustaining low prices without cause may prolong dependence on external funds.^16^ The key challenge for social programmes in pricing decisions is how to set prices that are low enough to be affordable to the clients and yet high enough to ensure sustainability of services.^16^ The potential range of prices may be quite broad, and there is usually no optimal price for a programme to charge.^17^

It is estimated that the first‐line regimen of antiretroviral treatment for adults and children costs about US$368 per person per year (about 1US$ per day).^18^ Adding 15%, which is the cost of logistics to the cost of drugs, translates approximately to US$393.75 (₦63 000) treatment cost per person per year and is multiplied eight times in the private sector, putting treatment beyond the reach for 97% of people in developing countries.^19^, ^20^ Analysing the financial sustainability options for ARVs leads to the question of affordability, as majority of people in the sub‐Saharan Africa live below the poverty line.^21^ With the increasing proportion of infected people and those on treatment, the need for increased funding arises as well as the possibility of donor fatigue. It has been reported that the increasing demand for antiretroviral treatment poses serious threat to sustainable funding for HIV/AIDS treatment,^22^ and there may come a time in future where the cost of treatment for HIV/AIDS would be borne by infected individuals or affected families, even if it be in part.

Presently, clinical management of HIV/AIDS requires lifelong treatment, and infected people would most likely commit to this if they perceive the benefits of treatment to outweigh the cost. An estimation of how much money an individual is willing to pay for goods/services gives an idea of how much value they attach to that product, although this is often limited to one's wealth.^23^ Contingent valuation is a method of valuing the benefits of an intervention in monetary terms based on revealed or stated consumer preferences expressed as maximum willingness to pay (WTP).^24^, ^25^, ^26^ CVM has been used as a survey‐based approach to elicit the value that people attach to health services by determining their maximum WTP for those services.^23^, ^27^, ^28^, ^29^ WTP is the maximum amount that an individual is ready to give up in order to use or consume particular goods or services.^30^ It is a useful technique for measuring the value that consumers place on goods and services by giving monetary values to the benefits.^31^, ^32^, ^33^ This has been used to elicit valid WTP amounts for health services in Nigeria and in other contexts.^34^, ^35^, ^36^, ^37^, ^38^

Although international funding allows patients to access antiretroviral treatment (ART) for free, assessment of patients WTP for ARVs will play a vital role in preparing an appropriate ART policy once external financial support becomes unavailable or limited.^7^ There is a dearth of evidence on how much people are willing to pay for HIV/AIDS treatment, especially in sub‐Saharan Africa where the epidemic is being felt the most. Available evidence, which dates back to 12 years, elicited WTP for services that the patients had not received or benefitted from.^39^ This study was conducted to evaluate the economic value expressed by clients receiving treatment for HIV/AIDS in a tertiary hospital using a contingent valuation method. It is unique in that it uses a questioning format that closely mimics price‐taking mechanism in the region^40^, ^41^ and additionally determines WTP for a household member.

This study provides evidence that could contribute to planning for sustainability of funding, to understand the values that the current beneficiaries attach to free ARVs, so as to determine its worthwhileness. Secondarily, it provides some information that is important for holistic funding of the treatment from all financing sources if donors withdraw or reduce their support—by estimating clients' WTP for ARVs in order to maintain lifelong therapy. WTP was elicited using the contingent valuation method (CVM) which has strong theoretical underpinning in welfare economics. The CVM can be used to monetize the values or monetize the benefits that people derive from or associate with services^42^ when markets do not exist or existing prices do not reflect the value of goods and services. As ARV drugs are provided free, market does not exist and it is important to know the extent to which people value the drugs.

## 2 STUDY METHODS

This study was conducted in south‐east Nigeria at the Federal Teaching Hospital Abakaliki (FETHA), Ebonyi state. FETHA is a public tertiary hospital that offers a variety of specialist and non‐specialist health services including prevention, treatment and control of communicable diseases. In collaboration with the Centre for Clinical Care and Research Nigeria, FETHA provides treatment services for HIV/AIDS clients through its centre for communicable disease control and AIDS relief. HIV/AIDS treatment services are provided by medical doctors and trained nurses. As at the time of this study, a total of 700 clients had enrolled and were receiving treatment from this centre. The centre runs a 4‐day clinic from Monday to Thursday and the daily patient load averages about 25.

### 2.1 Study population and recruitment procedure

The study population comprised of adults who were enrolled in and receiving treatment for HIV/AIDS from the communicable disease control and AIDS relief centre of the hospital during a 3‐week period in May 2014. An adult was defined as someone who was 18 years old or more. Sample size was determined using 95% confidence level, 5% precision and 29% as the proportion of clients who are willing to pay for HIV/AIDS treatment.^39^ A minimum sample size of 109 was calculated. The sample size was increased by 15% giving a total of 125 sample size to accommodate for non‐response. Participants were recruited consecutively as they exited the consulting room. We approached a total of 144 patients before we could get 125 who consented to be interviewed. Most refusals were due to disinterest or other time commitments. The participants represent the population of HIV‐infected people currently on treatment in Federal Teaching Hospital Abakaliki which is the largest HIV treatment centre in Ebonyi state. Non‐participants included HIV‐infected people <18 years of age, HIV‐infected adults who were not enrolled in treatment care, and HIV‐infected adults on treatment who did not have or keep to their clinic appointments in May 2014.

### 2.2 Data collection

A pre‐tested structured questionnaire was used to collect information from a sample of HIV/AIDS clients. The interviews were conducted by three undergraduate medical students who also participated in developing and pre‐testing the questionnaire. A 1‐day training was organized for them on quantitative interviewing using a WTP questionnaire. Role plays were performed in English and the local dialect. Clients were interviewed face‐to‐face as they exited the consulting room, using paper‐based questionnaires. Self‐ or interviewer‐administration of questionnaires was performed depending on client's literacy in English. Information was collected on respondent's demographic characteristics; awareness of ARV treatment procedures; ARV consumption patterns and expenditure; WTP for ARV drugs for HIV and AIDS; the amount they are willing to pay for themselves and for a member of their household. Information on household income, assets and food expenditure was collected.

#### 2.2.1 Eliciting willingness to pay

The two non‐market valuation methods (contingent valuation and discrete choice experiment) often provide statistically convergent WTP values, but we chose contingent valuation because it has advantage of face‐to‐face contact which reduces misunderstanding and could make spontaneous questions possible unlike DCE that lacks direct valuation question.^43^ CVM also has a stronger theoretical underpinning in welfare economics. The bidding game question format was used to elicit WTP. The bidding game technique is a distinct and standard contingent valuation question format. A comparative review of contingent valuation question formats for eliciting WTP was provided by Klose.^44^ These methods include the bidding game format which was closer for this study because it very closely mimics price‐taking mechanisms in the study area.^40^, ^41^ The bidding game format that was used in the study was modified so that participants have many bidding iterations that mimics price‐taking in markets in south‐east Nigeria, so that the most valid WTP estimates are elicited.^45^

Willingness to pay for self alone was elicited first, followed by WTP for an infected household member or spouse alone. In both cases, the respondents were first told that the price of monthly supply of ARVs for one person is ₦5000 (starting bid), and then asked whether they would be willing to pay for their own ARVs assuming they were not given free. The bidding game iteration that was used in the study was as follows:

1. The price of monthly supply of ARV is ₦5000, are you willing to pay? [ ] 1=yes (go to Q3), 0=no (go to Q2). 2=do not know (go to Q2).
2. What is the maximum amount you are willing to pay? [ ] (Interviewer: if more or equal to ₦4000 go to Q4, if less go to Q5).
3. What if the cost of ARV is ₦10 000 will you be willing to pay? 1=yes [ ], 0=no [ ] (no matter the answer, go to Q6)
4. What really is the maximum amount you are willing to pay for the ARV? [ ] (If more or equal to ₦5000 go to Q6, but if less than ₦5000 go to Q5).
5. The amount that you have quoted is too low, and cannot cover the cost of the drugs, and so you will have to increase the amount if you really want to be on treatment. So what is the final maximum amount you are willing to pay per month to continue your treatment? [₦ ]. (No matter the answer, go to Q6).
6. If due to inflation or other uncertainties, the cost of the ARV increases, what is the maximum amount you are very certain to pay bearing in mind your average monthly household income and money you spend on various items?[ ₦ ].

### 2.3 Data analysis

Frequencies and proportions were calculated for categorical variables while means were calculated for numeric variables. Households were grouped into four wealth quartiles namely poorest, very poor, poor and least poor, and this was calculated using principal component analysis based on household asset ownership (refrigerator, radio, television, bicycle, car, etc.) and weekly food expenditure per capita. Demographic characteristics and wealth quartiles were cross‐tabulated with participants' WTP to determine associations. Appropriate statistical tests of association were performed at 95% confidence. The mean WTP amounts, for both respondents WTP for self and for others, respectively, were computed from the final stated WTP amounts to the last question on the bidding game iteration. The theoretical validity of the elicited respondents' WTP for self and for others was assessed using ordinary least squares (OLS) multiple regression analysis. The dependent variables were mean WTP and some variables that were hypothesized to explain WTP were the independent variables (Table 1). The independent variables reflect the demographic characteristics of respondents, their experiences with receiving care (additional cost, waiting time, etc.) and household socio‐economic status. Demographic variables such as education, age, employment status and income have been reported to influence WTP as well as other factors that constitute stress.^23^, ^28^, ^46^, ^47^ Currency exchange rate of US$1=₦160 as at 30/04/2014 was used.

**Table 1:** Description of independent variables hypothesized to explain willingness to pay (WTP) for antiretroviral drugs

Variables	Explanation	Measurement	Hypothesized relationship with WTP
Age of respondent	How old (in years) the respondents are	A continuous quantitative measurement	The older the respondent, the less the WTP
Relationship status of respondent	Whether respondents have a spouse	1=have a spouse 0=do not have a spouse	Respondents with spouses will have higher WTP than others
Gender of respondent	Whether respondents are male or female	1=male 0=female	Males will have higher WTP than females
Education	Whether respondents had formal education	1=had formal education 0=had no formal education	People with formal education will have higher WTP than others
Employment status	Whether the respondent is employed	1=employed 0=unemployed	People who are employed will have higher WTP than others
Knowledge of risks of non‐adherence	Respondents know at least one risk of not adhering to treatment protocol	1=know risk of compliance 0=do not know risk of compliance	Those who know the risk of non‐adherence will have higher WTP than others
Pays transport cost	How much respondents have to pay for transport per clinic visit	A continuous quantitative measurement	The higher the transport cost the lower the WTP
Average monthly income of spouse	Average monthly income of respondents' spouses from all sources	A continuous quantitative measurement	The higher the spouses' income, the higher the WTP
Socio‐economic status (SES)	An index of the socio‐economic status of the respondents' households	A continuous quantitative measurement	The higher the SES, the higher the WTP

### 2.4 Ethical considerations

This research proposal and consent procedure were approved by a Health Research and Ethics Committee, and the registration number is FETHA/HREC/22/04/2014. Participants were informed of the purpose of the study, their roles, benefits and rights as participants, and possible risks from participation. Verbal informed consent was obtained from all participants, and their anonymity was maintained by non‐inclusion of self‐identifiers in the questionnaire. Participant consent was documented using an informed consent form with provision for ticking (√) in a “yes” or “no” box. The date of obtaining consent was also documented in the form. Verbal consent was preferred to written consent because some of the respondents did not want to append a signature or any form of identification on the form. Participants were interviewed singly in empty consulting rooms to ensure their privacy.

## 3 RESULTS

A total of 144 clients were approached for interview, 125 consented and were interviewed, but 123 questionnaires were appropriate for analysis.

The participants had a mean age of 32.29 (±7.2) years. Other demographic characteristics are shown in Table 2. Most of the respondents, 56 (45.5%), were within the age range of 26‐35 years. There were more female participants 66 (53.7%) than males, and there were more married people, 79 (64.2%), than unmarried. Majority of the participants had some form of education and 50 (40.7%) of them had at least primary education.

**Table 2:** Socio‐demographic characteristics of respondents

Variables	Frequency, n (%)
Age category (y)	
15‐25	25 (20.3)
26‐35	56 (45.5)
36‐45	39 (31.7)
>45	3 (2.4)
Marital status	
Married	79 (64.2)
Single	44 (35.8)
Gender	
Male	57 (46.3)
Female	66 (53.7)
Educational status	
Primary	50 (40.7)
Secondary	46 (37.4)
Tertiary	16 (13.0)
None	11 (8.9)
Employment status^a^	
Paid employment	29 (23.6)
Self‐employed	77 (62.6)
Unemployed	17 (13.8)
SES	
Poorest	31 (25.2)
Very poor	31 (25.2)
Poor	33 (26.8)
Least poor	28 (22.8)

a Paid employment covers people who are employed by government and organized private sector and earn monthly salaries; self‐employed refers to those who have their own businesses or enterprise or who generate their own income.

A total of 120 (97.6%) of the respondents stated they knew the price of a monthly supply of their ARVs. As shown in Table 3 41 (33.3%) of the respondents were willing to pay the first bid of US$31.25 monthly for themselves. Of the 67 respondents who had a household member or spouse living with HIV/AIDS, 23 (34.3%) were willing to pay the first bid of US$31.25 for their monthly supply of ARVs.

**Table 3:** Willingness to pay (WTP) for antiretroviral drugs for self and household member/spouse

Variables	Measurement (N=123) Frequency (%)
Willing to pay first bid of ₦5000 (US$31.25) for self	41 (33.3)
Willing to pay ₦5000 (US$31.25) first bid for household member/spouse	23 (34.3)^a^

Mean WTP	Naira (₦)	US$
Mean WTP self	2452.33 (±2175.63)	15.32 (±13.59)
Minimum‐Maximum WTP self	200‐15 000	1.25‐93.75
Mean WTP for others	2441.94 (±1912.30)	15.26 (±11.95)
Minimum‐Maximum WTP others	200‐10 000	1.25‐166.67

a N=67, which is number of participants with household member living with HIV/AIDS.

Table 3 also shows respondents' mean WTP for monthly supply of ARVs for HIV/AIDS for themselves and their spouses or a household member. Their final maximum WTP for self was US$15.32 ±13.59 for themselves and US$15.26 ±11.95 for a member of their household.

Associations between participants WTP ₦5000 (US$31.25) and ₦10 000 (US$62.5) for ARVs for themselves and a household member or spouse are presented in Table 4. Whole numbers refer to the people in the demographic categories who expressed positive WTP, and the proportions refer to the percentage of people in that category who expressed positive WTP. Statistical significant association was found between participants' WTP ₦5000 (US$31.25) for their monthly supply of ARVs and their occupation; traders and civil servants expressed more WTP than other occupations (*P*<.05). Participants in the higher wealth quartiles were more willing to pay ₦5000 (US$31.25) for their monthly supply of ARVs for HIV/AIDS than those in the lower quartiles (*P*<.001).

**Table 4:** Willingness to pay (WTP) for antiretroviral drugs by different population groups

Variables	WTP for self		WTP for household member	
	₦5000 (US$31.25)	₦10 000 (US$62.5)	₦5000 (US$31.25)	₦10 000 (US$62.5)
Age category (y)				
15‐25	10 (40)	2 (8)	3 (30)	0
26‐35	19 (33.9)	10 (17.9)	14 (45.2)	4 (12.9)
36‐45	11 (28.2)	2 (5.1)	5 (20.8)	1 (4.2)
>45	1 (33.3)	0	1 (50)	0
χ^2^ (*P*‐value)	0.97 (.81)	4.51 (.21)	3.85 (.27)	2.19 (.53)
Gender				
Male	25 (43.9)	6 (10.5)	13 (37.1)	3 (6.2)
Female	16 (24.2)	8 (12.1)	10 (31.2)	2 (8.6)
χ^2^ (*P*‐value)	1.32 (.25)	0.08 (.78)	0.28 (.61)	0.13 (.72)
Marital status				
Married	25 (31.6)	7 (8.9)	19 (33.3)	4 (7.0)
Unmarried	16 (36.4)	7 (15.9)	4 (40.0)	1 (10.0)
χ^2^ (*P*‐value)	0.28 (.59)	1.39 (.25)	0.17 (.68)	0.11 (.74)
Level of education				
Primary	14 (28)	5 (10)	12 (35.3)	1 (2.9)
Secondary	17 (37)	4 (8.7)	5 (25)	1 (5.0)
Tertiary	8 (50)	4 (25)	6 (66.7)	3 (33.3)
None	2 (18.2)	1 (8.3)	0	0
χ^2^ (*P*‐value)	4.05 (.25)	3.42 (.33)	6.28 (.08)	6.86 (.05)
Employment status				
Paid employment	15 (51.7)	7 (24.1)	10 (55.6)	4 (22.2)
Self‐employed	20 (26.0)	6 (7.8)	13 (26.5)	1 (2.0)
Unemployed	6 (35.3)	1 (5.9)	0	0
χ^2^ (*P*‐value)	6.32 (.04)	6.12 (.04)	4.92 (.04)	7.76 (.02)
Wealth index				
Poorest	6 (19.3)	1 (3.2)	3 (16.7)	1 (5.6)
Very poor	4 (12.9)	3 (9.4)	4 (28.6)	0
Poor	14 (42.4)	3 (9.1)	4 (25)	0
Least poor	17 (60.7)	7 (25)	12 (63.2)	4 (21.1)
χ^2^ (*P*‐value)	25.53 (<.01)	7.45 (.05)	9.65 (.02)	5.32 (.07)

Whole numbers refer to the people in the demographic categories expressed positive WTP. The proportions refer to the percentage of people in that category who expressed positive WTP.

### 3.1 OLS regression analysis

Table 5 shows the full and reduced OLS regressions for WTP for the respondents and a member of their household. Among the statistically significant variables, it strikingly shows that WTP was positively related to average monthly income and knowledge of risk of adherence for both WTP for self and household member. It was also positively associated with employment status for both WTP for self (reduced model) and household member, and socio‐economic status for WTP for self alone. WTP was negatively related to age and paying for transport showing that older age and higher transport cost led to decreased WTP.

**Table 5:** Full and reduced ordinary least squares models of willingness to pay (WTP) for antiretroviral drugs for self and for household member

Independent variables	WTP for self (N=123)		WTP for others householders (N=123)	
	Full model coefficient (SE)	Reduced model coefficient (SE)	Full model coefficient (SE)	Reduced model coefficient (SE)
Age	−1.73 (1.66)	−1.90 (1.67)*	−1.83 (1.49)*	−1.86 (1.48)**
Male	2.96 (2.77)*	3.01 (2.78)*	2.79 (2.63)	2.78 (2.63)
Married	−3.16 (2.83)**	−3.18 (2.83)**	−1.65 (2.89)	—
Education	2.42 (3.18)	—	2.94 (3.04)	—
Employment status	2.72 (2.86)	3.18 (2.77)***	3.08 (2.83)*	3.15 (2.78)**
Knowledge of risks of non‐adherence	3.28 (2.88)**	3.41 (2.87)***	3.12 (2.79)**	3.10 (2.79)**
Pays transport cost	−2.69 (2.85)	—	−3.08 (2.72)**	−3.10 (2.72)**
Average monthly income	0.01 (0.02)**	0.06 (0.02)**	0.037 (0.01)***	0.04 (0.01)***
Socio‐economic status	3.06 (2.57)***	2.98 (2.54)***	2.29 (2.46)	2.51 (2.34)
*F* statistics	5.29***	7.11***	3.96***	5.32***
Adjusted *R* ^2^	.308	.323	.317	.337

*P<.10; **P<.05; ***P<.01.

## 4 DISCUSSION

This study examined whether people with HIV/AIDS who have been receiving treatment free of charge would be willing to pay for their ARVs and how much they are willing to pay should the need arise. Having told them how much it would cost to purchase their monthly supply of ARVs, one‐third of our study participants expressed WTP for their own drugs. When asked whether they would be willing to pay the same amount for an infected household member or spouse to get their monthly supply of drugs, about one‐third said they would. The regression analysis showed that the elicited WTP values were construct‐valid. In addition, there was no evidence of starting‐point bias because there was no clustering of WTP value around the starting bid.

Although over 90% of our participants knew the cost of ARVs for HIV/AIDS, their maximum mean WTP for a monthly supply of these drugs was less than half of the actual cost of the drugs, implying that knowledge of the cost of ARVs does not necessarily translate into WTP, rather WTP is subject to socio‐economic status of participants. Thus, the knowledge of cost of ARV does not have anchor effect or starting‐point bias because if the participant's bid reflects his or her true value of the good or service, then it should not matter what initial amount was used to begin the bidding game.^48^, ^49^, ^50^ As a matter of fact, ART is a “normal good” for which its demand increases with assets and negatively related to price—as prices go up, demand will come down.^29^ Studies indicate that WTP is significantly correlated with education, marital status, living area and distance from house to clinic, monthly income of both the patient and their household, household economic condition and monthly expenditure.^3^, ^28^, ^38^, ^51^, ^52^

It was found that being willing to pay ₦5000 (US$31.25) per month for ARVs had a positive statistical significant association with employment status of clients. Those who had more regular sources of income (paid employment) expressed more WTP than those whose income sources were unreliable (self‐employed) probably because in order to be able to give up a certain amount of money on a regular basis, an individual needs to be clear about how much they earn and how often their earnings come in. People who have steady sources of income are more able to make regular payments than people who are uncertain when the next money will come and how much that would be. Thus, confirming Nguyen et al. that individual capacity to pay for antiretroviral drug is strongly influenced by socio‐economic factors such as monthly income, household economic condition and marital status. Indeed, as socio‐economic status increases, level of WTP also increases.^28^, ^46^, ^52^ Participants' socio‐economic status was significantly associated with their WTP for ARVs. Those in the higher wealth quartiles were more willing to pay ₦5000 (US$31.25) for a monthly supply of their drugs than those in the lower quartiles. In the same vein, the married were more willing to pay for ARVs than those who were not married, because patients living with a spouse could share the expenditure with their partner.^3^

Considering that only 51.8% of Nigerians are employed and that civil servants made up less than a quarter of people receiving treatment for HIV/AIDS in our study site, we can conclude that over three quarters of people who are infected with HIV/AIDS may not be able to regularly purchase their ARVs if they had to do so. As one of the major contributing factors to this inability to pay was the patients' unemployment situation, which leads to low, and often unstable, incomes.^3^, ^53^ Moreover, medical costs associated with HIV/AIDS treatment are likely to impose a significant financial burden on patients without any support from government or other funding sources. In order to access and maintain treatment, patients have to incur many nonmedical costs, including travel costs, meals, accommodation, opportunity costs and other medications. Therefore, even without the need to pay for ART itself, many patients face a significant financial burden just to access and sustain their treatment.^3^, ^54^ Potential solutions to cope with household income to finance health care for poorer households have been explored such as mutual health organizations and microfinancing for health.

A limitation of the study was that hypothetical WTP for ARVs was elicited which may differ from revealed preferences through actual observations of how much is paid. It has been argued that stated preferences when compared to revealed preferences may produce under‐ or overestimates depending on the social and economic contexts at the time. Willingness to pay surveys allow programme managers to simulate price‐related changes in demand without actually changing prices, thereby helping them to make decisions based on empirical information.^29^ Secondly, the use value in an ex‐post perspective of a population who already have access to free drugs reduces the policy relevance of the results. Additional investigation of option value could have added benefits. Thirdly, the bidding iteration used does not follow the typical bidding algorithm and may have induced responses which may not be a true reflection of the value of ARVs to users. Finally, the quality of life and illness severity of the respondents were not measured, because the factors could have affected their levels of WTP for ARVs. This is because it has been found that bad health outcomes may explain some differences in WTP and some authors found that a low quality of life is correlated with no desire to receive health care to prolong life.^55^ These factors should be explored in future WTP for ARV studies. The strength of this study is that it elicits WTP using a bidding game iteration that is contextually valid and mimics price‐taking mechanism in the study area. While other studies limit their WTP questions to the respondents alone, ours additionally elicits WTP values for a household member.

## 5 CONCLUSION

Only a third of clients receiving treatment for HIV/AIDS are willing to pay for their ARVs and that of a family member or spouse. Their mean WTP for a monthly supply of ARVs for HIV/AIDS falls below the estimated cost of the drugs. Regular source of income and higher socio‐economic status are significantly associated with WTP of US$31.25 per month to purchase ARVs for HIV/AIDS. As less than a quarter of the clients surveyed have regular sources of income, charging user fees for HIV/AIDS treatment could lead to financial inaccessibility for most infected people. Governments need to consider and put in place more sustainable measures to ensure stable funding for prevention and control of HIV/AIDS. A solution that has been suggested is a co‐payment system for ART. However, in order for such a system to be effective, patients must be willing to pay for their share of services received. The policy implications of this study indicate that to get a greater number of individuals on ART government would have to find other mechanisms of lowering the price of the drugs or subsidizing treatment cost for HIV/AIDS patients.

## CONFLICTS OF INTEREST

The authors declare that they have no conflict of interests.
